# Soluble HLA-G and HLA-E Levels in Bone Marrow Plasma Samples Are Related to Disease Stage in Neuroblastoma Patients

**DOI:** 10.1155/2016/7465741

**Published:** 2016-08-16

**Authors:** Fabio Morandi, Sarah Pozzi, Barbara Carlini, Loredana Amoroso, Vito Pistoia, Maria Valeria Corrias

**Affiliations:** ^1^Laboratorio di Oncologia, Istituto Giannina Gaslini, Via Gaslini 5, 16147 Genoa, Italy; ^2^Centro Cellule Staminali, IRCCS AOU San Martino-IST, Largo R. Benzi 10, 16132 Genoa, Italy; ^3^UOC Oncologia, Istituto Giannina Gaslini, Via Gaslini 5, 16147 Genoa, Italy

## Abstract

The role of nonclassical HLA-class Ib molecules HLA-G and HLA-E in the progression of Neuroblastoma (NB), the most common pediatric extracranial solid tumor, has been characterized in the last years. Since BM infiltration by NB cells is an adverse prognostic factor, we have here analyzed for the first time the concentration of soluble (s)HLA-G and HLA-E in bone marrow (BM) plasma samples from NB patients at diagnosis and healthy donors. sHLA-G and sHLA-E are present in BM plasma samples, and their levels were similar between NB patients and controls, thus suggesting that these molecules are physiologically released by resident or stromal BM cell populations. This hypothesis was supported by the finding that sHLA-G and sHLA-E levels did not correlate with BM infiltration and other adverse prognostic factors (*MYCN* amplification and age at diagnosis). In contrast, BM plasma levels of both molecules were higher in patients with metastatic disease than in patients with localized NB, thus suggesting that concentration of these molecules might be correlated with disease progression. The prognostic role of sHLA-G and sHLA-E concentration in the BM plasma for NB patients will be evaluated in future studies, by analyzing the clinical outcome of the same NB patients at follow-up.

## 1. Introduction

Neuroblastoma (NB) is the most common extracranial solid tumor in children, with an incidence of 1 case per 100.000 children per year, and causes 15% of cancer deaths in pediatric age. NB originates from the sympathetic nervous system, most frequently in the adrenal medulla or the paraspinal ganglia. The causes are unknown, although 1-2% of NB may have a hereditary basis. Different genetic alterations have been characterized in NB, that is, gain-of-function of* ALK* gene, losses of 11q and 1p, gain of 17q, and amplification of the* MYCN* gene. NB is heterogeneous, as it may undergo spontaneous remission or evolve to progressive metastatic disease, with dissemination to lymph nodes, bone, bone marrow, liver, skin, and other organs [1]. In particular, BM infiltration is an indicator of poor outcome for NB patients [2]. The International Neuroblastoma Risk Group staging system takes into account genetic alterations, DNA ploidy, histological features, and clinical data, as criteria for defining the risk classes. The prognosis of low/intermediate risk NB patients is favorable, and tumors can be cured by surgery alone or minimal chemotherapy. In contrast, high-risk NB patients' prognosis is poor, in spite of aggressive treatment based on surgery, chemotherapy, radiation therapy, hematopoietic stem cell transplantation, and adjuvant therapy with retinoic acid. In fact, survival rates of these patients at 5 years are less than 50% [3].

In the last years, the role of HLA-class Ib molecules in the progression of NB has been characterized by our group [4–7] and by others [8]. HLA-Ib family includes HLA-G, HLA-E, HLA-F, and HLA-H. In contrast with high polymorphic HLA-Ia molecules (HLA-A, HLA-B, and HLA-C) all these molecules display a limited polymorphism, with few alleles encoding a limited number of functional proteins. Moreover, although HLA-class Ib molecules can bind small peptides and present them to specific CD8^+^ T cell subsets (similarly to HLA-class Ia counterparts), their main function is the modulation of the immune response in both physiological and pathological conditions [9]. HLA-G and HLA-E are the best characterized among HLA-Ib molecules. HLA-G has seven different isoforms, four membrane bound (namely, HLA-G1, HLA-G2, HLA-G3, and HLA-G4) and three soluble (namely, HLA-G5, HLA-G6, and HLA-G7), that are generated by alternative splicing from the same primary transcript. HLA-G can interact with at least four receptors, namely, immunoglobulin-like transcript (ILT)2, ILT4, KIR2DL4, and CD160, thus affecting the function of different immune effector cells (T and B lymphocytes, natural killer NK cells, dendritic cells, granulocytes, and monocytes) [10]. In contrast, HLA-E can be expressed as membrane bound or soluble isoform (generated through metalloproteases cleavage) and can inhibit CD8^+^ T cells or NK cells though interaction with CD94/NKG2A heterodimeric receptor. However, HLA-E can also interact with the activating receptor CD94/NKG2C, thus leading to NK cell activation. These interactions are crucial during trophoblast implantation to abrogate NK cell lysis of semiallogeneic fetal tissue and, on the other hand, to activate NK cell functions in the process of tissue remodeling [11].

We have previously demonstrated that soluble (s)HLA-G concentration is higher in plasma samples from NB patients than in controls, and sHLA-G can be released by NB cells themselves, or by monocytes (stimulated by soluble factors secreted by tumor cells). Moreover, high sHLA-G plasma levels correlated with NB patients' relapse [4]. Finally, we have assessed that HLA-G is expressed by metastatic NB cells in the bone marrow from NB patients [6].

Also soluble HLA-E levels are higher in NB patients than in healthy controls. However, we have demonstrated that high plasma levels of sHLA-E at diagnosis correlated with a better overall survival (OS) of NB patients at follow-up, in contrast with sHLA-G [5].

Here, we demonstrated for the first time that sHLA-G and sHLA-E are present also in BM plasma samples derived from either NB patients at diagnosis or healthy donors. Moreover, we have assessed that sHLA-G and sHLA-E levels in BM plasma samples are related to the stage of the disease. Analysis of these patients at follow-up will reveal whether sHLA-G and sHLA-E concentration in BM plasma may predict the clinical outcome of NB patients.

## 2. Materials and Methods

### 2.1. Patients and Controls

The study was approved by the Ethics Committee of the G. Gaslini Institute, Genoa, Italy.

NB patients (*n* = 31) were diagnosed during 2016 in AIEOP centers. Bone marrow (BM) samples were collected at diagnosis and centralized at Istituto Giannina Gaslini in Genoa, Italy. BM plasma samples were obtained after centrifugation (3000*g* × 10′). NB patients were staged according to the International Neuroblastoma Staging System [12]. Patients' characteristics, that is, age at diagnosis, sex,* MYCN* amplification (single copy or amplified), BM infiltration, and stage, are summarized in Table 1.

As controls, BM aspirates were obtained from 13 healthy donors, selected according to the Transplant Unit Clinical Protocol of Ematologia 2 at the IRCCS San Martino-IST in Genoa, following a written informed consent at the time of donation. Samples were processed as described in [13], and an aliquot was taken at the end of processing to perform quality control tests, such as CD34^+^ cell count, in vitro progenitors' cell growth, and sterility. The remaining BM blood sample from this aliquot was subjected to centrifugation (3000*g* × 10′) to obtain BM plasma. Donor's characteristics are summarized in Table 2.

All BM plasma samples were stored at −80°C until use.

### 2.2. ELISA

Enzyme-Linked Immunosorbent Assay (ELISA) for sHLA-G and sHLA-E was performed as previously described [14]. Briefly, MaxiSorp Nunc-Immuno 96-microwell plates (Nunc A/S, Roskilde, Denmark) were coated overnight at 4°C with 1 *µ*g/mL of MEM-G9, specific for HLA-G HC (Exbio, Prague), that recognizes sHLA-G1/G5, or 3D12 mAb, specific for HLA-E HC (eBioscience, Science Center Drive, San Diego, CA, USA). After three washes with PBS 0.05% Tween 20 (washing buffer), plates were saturated with 200 *μ*L/w of PBS 2% BSA (Sigma, St. Louis, MO, USA) for 30 min at RT. One hundred *μ*L of BM plasma samples and serial dilutions of 721.221.G1 cell line supernatant (for HLA-G) or total extract from normal peripheral blood mononuclear cells (standard) were added to each well and incubated at RT for 1 hour. After three washes, 100 *μ*L of detection reagent (HRP-conjugated anti-*β*2 microglobulin mAb, Exbio, Vestec, CZ) was added, and plates were incubated for 1 hour at RT. After three washes, 100 *μ*L of TMB (substrate for HRP, Sigma) was added, and reaction was stopped after approximately 30 minutes by adding H_2_SO_4_ 5 N. Absorbance at 450 nm was measured using Infinite® 200 PRO spectrometer (Tecan Group Ltd., Seestrasse, Männedorf, Switzerland). Results are expressed as ng/mL sHLA-G and arbitrary units/mL sHLA-E (1 unit = quantity of sHLA-E in 1 *µ*g of total extract).

### 2.3. Statistics

Normal distribution of data was tested using Kolmogorov-Smirnov test, using Prism software (GraphPad Software Inc., La Jolla, CA). Since data distribution was not normal, differences in plasma levels between (i) patients and controls or (ii) different groups of patients were compared by Mann-Whitney test, using Prism software. Correlations between plasma levels of sHLA-G and sHLA-E were calculated by Spearman's test using Prism software. A *p* value 0.05 was considered as statistically significant. Significance ranges are the following: ^*∗*^ *p* < 0.05; ^*∗∗*^ *p* < 0.01; and ^*∗∗∗*^ *p* < 0.001.

## 3. Results

### 3.1. Soluble HLA-Ib Molecules Are Physiologically Present in BM Plasma Samples

**Figure 1:**
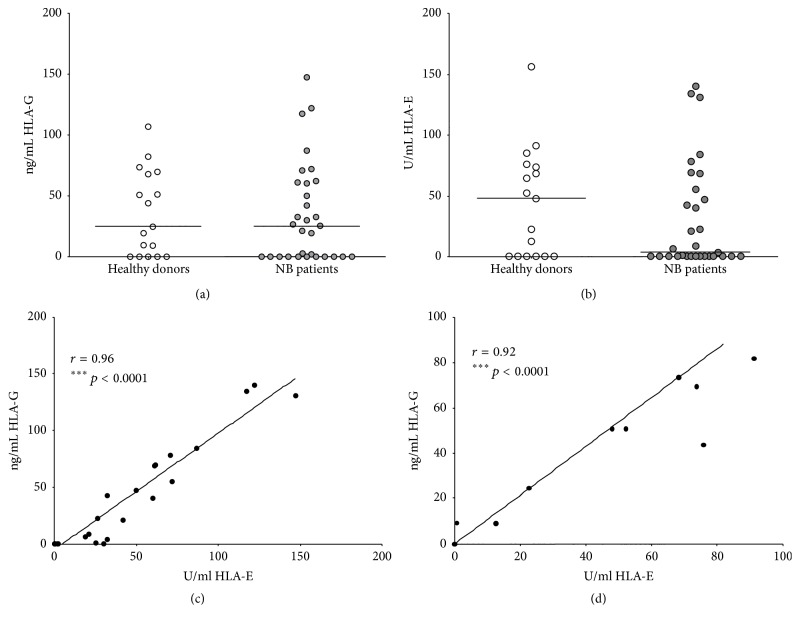
Levels of soluble (s)HLA-G (a) and sHLA-E (b) have been analyzed in BM plasma samples from NB patients (grey circles) and healthy BM donors (white circles). Horizontal lines indicated medians. Results are expressed as ng/ml (sHLA-G) or arbitrary units (U)/ml (sHLA-E). Correlation between BM plasma levels of sHLA-G and sHLA-E have been analyzed in NB patients (c) and controls (d). Linear regression of data and* r* and* p* values are indicated.

First, we have tested sHLA-G and sHLA-E concentration in BM plasma samples from NB patients and healthy donors. As shown in Figure 1(a), sHLA-G concentration was similar between NB patients (median ± SE: 24.69 ± 8.45 ng/mL) and controls (25.16 ± 7.38 ng/mL). In contrast, sHLA-E levels were lower in NB patients (3.72 ± 7.89 U/mL) than in controls (48.01 ± 10.93 U/mL). However, such difference was not statistically significant, likely due to the wide distribution of the results in both groups (Figure 1(b)). Finally, sHLA-G and sHLA-E levels in BM plasma samples from NB patients (*r* = 0.96, *p* < 0.0001, Figure 1(c)) and healthy donors (*r* = 0.92, *p* < 0.0001, Figure 1(d)) strongly correlated with each other.

### 3.2. Soluble HLA-Ib Molecules Correlated with Disease Progression

We have next analyzed possible correlation between sHLA-G and sHLA-E levels in BM plasma samples and patient's characteristics or clinical parameters. Accordingly, NB patients were divided into two groups on the basis of (i)* MYCN* amplification (single copy* versus* amplified), (ii) BM infiltration (not infiltrated* versus* infiltrated), (iii) age at diagnosis (<18 months* versus* >18 months), (iv) stage of the disease (stages 1-2* versus* stages 3-4), and (v) sex (male* versus* female). Next, differences in sHLA-G and sHLA-E levels between these groups of NB patients have been evaluated.

**Figure 2:**
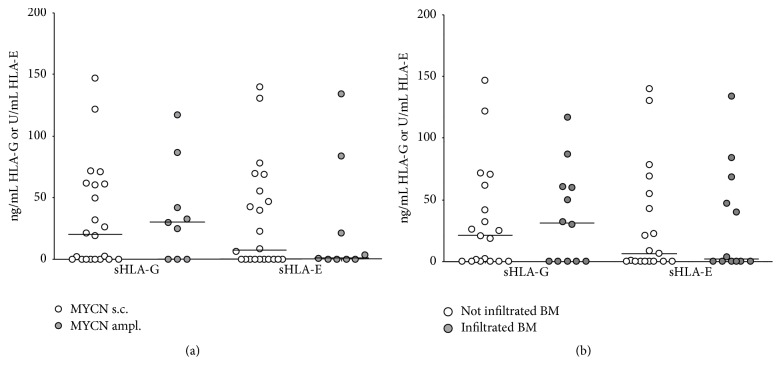
Levels of soluble (s)HLA-G and sHLA-E in BM plasma samples have been analyzed in patients presenting (grey circles) or not (white circles) MYCN amplification (a) or BM infiltration (b). Horizontal lines indicated medians. Results are expressed as ng/ml (sHLA-G) or arbitrary units (U)/ml (sHLA-E).

No significant differences in sHLA-G levels have been detected between NB patients (i) carrying amplified (29.96 ± 13.57 ng/mL) or single-copy (23.65 ± 9.6 ng/mL)* MYCN* gene (Figure 2(a)) and (ii) presenting (31.17 ± 11.99 ng/mL) or not (21.05 ± 9.91 ng/mL) NB cells infiltrating the BM (Figure 2(b)). In contrast, sHLA-E levels were higher in (i) patients with single-copy* MYCN* (7.56 ± 9.17 U/mL) than in those with amplified* MYCN* (1.03 ± 16.2 U/mL) (Figure 2(a)) and in (ii) patients with infiltrated BM (6.45 ± 10.29 U/mL) than in those without BM infiltration (1.86 ± 12.43 U/mL) (Figure 2(b)). However, such differences were not statistically significant.

**Figure 3:**
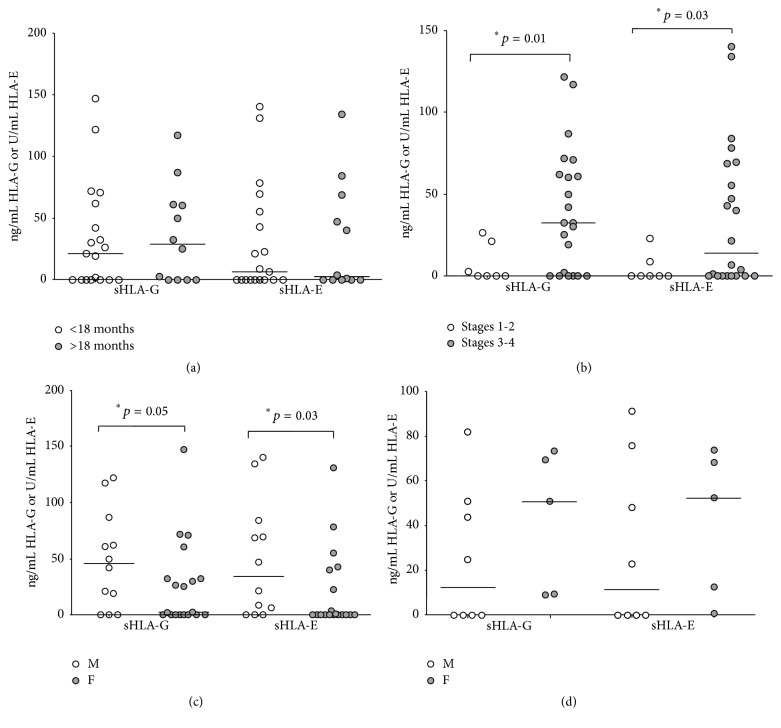
Levels of soluble (s)HLA-G and sHLA-E in BM plasma samples have been analyzed in patients with age at diagnosis above (grey circles) or below (white circles) 18 months (a) and in patients with stages 3-4 (grey circles) or 1-2 (white circles) disease (b). Differences between female (grey circles) or male (white circles) subjects have been analyzed in NB patients (c) and healthy donors (d). Horizontal lines indicated medians. Results are expressed as ng/ml (sHLA-G) or arbitrary units (U)/ml (sHLA-E).* p* values are indicated where differences are statistically significant.

Furthermore, both sHLA-G and sHLA-E levels were similar between patients with an age below (21.05 ± 9.92 ng/mL sHLA-G and 6.45 ± 10.29 U/mL sHLA-E) or above (28.77 ± 11.36 ng/mL sHLA-G and 2.37 ± 12.81 U/mL sHLA-E) 18 months at diagnosis (Figure 3(a)). Notably, no correlation was found between age and sHLA-G or sHLA-E levels in BM plasma samples in healthy donors (data not shown).

Both sHLA-G and sHLA-E levels were significantly higher in patients with disease stages 3-4 (32.34 ± 8.08 ng/mL sHLA-G and 13.87 ± 9.42 U/mL sHLA-E) than in those with disease stages 1-2 (0 ± 4.32 ng/mL sHLA-G and 0 ± 3.27 U/mL sHLA-E, *p* = 0.01 and 0.03, resp.) (Figure 3(b)). Surprisingly, both sHLA-G and sHLA-E levels were found to be higher in male (45.87 ± 12.5 ng/mL sHLA-G and 34.19 ± 14.83 U/mL sHLA-E) than in female (2.52 ± 8.81 ng/mL sHLA-G and 0 ± 8.18 U/mL sHLA-E, *p* = 0.05 and 0.03, resp.) NB patients (Figure 3(c)). In contrast, healthy donors showed higher levels of sHLA-G and sHLA-E in female (50.74 ± 14.1 ng/mL sHLA-G and 52.25 ± 14.8 U/mL sHLA-E) than in male (12.35 ± 10.98 ng/mL sHLA-G and 11.33 ± 13.25 U/mL sHLA-E) subjects. However, such differences were not statistically significant (Figure 3(d)).

## 4. Discussion

To the best of our knowledge, this is the first demonstration of the presence of sHLA-class Ib molecules HLA-G and HLA-E in BM plasma samples. Previous studies have demonstrated that sHLA-G can be released by some cell populations that are present in the BM environment, such as erythroblasts [15] and mesenchymal stromal cells [16–19]. In contrast, no information is available regarding HLA-E expression and release in the BM. The strong correlation observed between the levels of these two molecules in BM samples either from NB patients or controls suggested that both molecules may be released by the same cell populations, or at least induced by similar* stimuli*.

We have previously demonstrated that metastatic NB cells in the BM expressed high levels of HLA-G on their surface, in contrast with primary tumors, that tested negative for HLA-G [6]. Here, we have demonstrated that both sHLA-G and sHLA-E are present at similar levels in NB patients and healthy donors, thus suggesting that malignant metastatic NB cells are unlikely involved in their release. This observation is further confirmed by the finding that BM infiltration by metastatic NB cells did not affect sHLA-G or sHLA-E levels in BM plasma samples. Moreover,* MYCN* amplification and age at diagnosis that represent important prognostic factors were not related to sHLA-G and sHLA-E levels in BM, thus further suggesting that these molecules might be released by BM stromal cells or BM resident cell populations instead of NB cells themselves, and may be present in the BM environment in physiological conditions. However, the increased tumor burden might be correlated to a higher release of tumor-derived factor(s) that, in turn, can upregulate HLA-G and HLA-E production by BM stromal cells.

The finding that sHLA-G and sHLA-E BM plasma levels are higher in male than in female patients is in line with a previous study on multiple sclerosis, where the authors demonstrated that sHLA-G levels in plasma samples were higher in male than in female patients [20]. However, this study has been carried out using peripheral blood plasma samples, and this is the first demonstration of this difference between male and female subjects in bone marrow plasma samples. Notably, such difference may be a prerogative of NB patients, since sHLA-G and sHLA-E levels were higher in female than in male normal subjects.

The most important finding of our study is the demonstration that sHLA-G and sHLA-E levels were significantly higher in BM plasma samples from patients with metastatic disease than in patients with localized NB. This data may suggest that the levels of these molecules in the BM at diagnosis might be associated with disease progression and might be predictive of the clinical course of NB patients. However, this hypothesis can be confirmed only by analyzing the clinical parameters of these patients at follow-up.

## 5. Conclusions

In conclusion, we demonstrated for the first time that soluble HLA-Ib molecules HLA-G and HLA-E are present in BM plasma samples in physiological and pathological conditions, and their concentration correlated with stage disease in NB patients. The prognostic value of sHLA-G and sHLA-E concentration in BM plasma samples from NB patients at diagnosis has to be confirmed in future studies.

## Figures and Tables

**Table 1:** Neuroblastoma patients' characteristics. First row indicates all the variables analyzed in NB patients, second row indicates the subgroups for each variable, and third row indicates the number of subjects in each group.

Age at diagnosis (months)		Sex		MYCN		BM infiltration				Stage				
<18	>18	M	F	s.c.	Ampl.	Neg.	1+	2+	3+	1	2	3	4	4s
19	12	12	19	22	9	19	5	4	3	6	1	10	12	2

s.c.: single copy; Ampl.: amplified; Neg.: negative.

**Table 2:** Healthy donors' characteristics. First row indicates all the variables analyzed in healthy controls, second row indicates the subgroups for each variable, and third row indicates the number of subjects in each group.

Age (years)		Sex	
Range	Mean ± SD	M	F
20–54	39.6 ± 13	8	5
